# Rehabilitation status and preference for medical choice behaviours of long COVID-19 in China: a national cross-sectional study and discrete choice experiment

**DOI:** 10.7189/jogh.16.04081

**Published:** 2026-03-20

**Authors:** Jie Deng, Liyuan Tao, Nan Liu, Jun Li, Chenyuan Qin, Wenxin Yan, Yaping Wang, Min Du, Qiao Liu, Jue Liu

**Affiliations:** 1 Department of Epidemiology and Biostatistics, School of Public Health, Peking University, Beijing, China; 2 Peking University First Hospital, Beijing, China; 3 Peking University Health Emergency Management Center, Beijing, China; 4 Clinical Epidemiology Research Center, Peking University Third Hospital, Beijing, China; 5 Department of Rehabilitation Medicine, Peking University Third Hospital, Beijing, China; 6 School of Health Humanities, Peking University, Beijing, China; 7 Vanke School of Public Health, Tsinghua University, Beijing, China; 8 Key Laboratory of Epidemiology of Major Diseases (Peking University), Ministry of Education, Beijing, China; 9 Institute for Global Health and Development, Peking University, Beijing, China

## Abstract

**Background:**

Long COVID-19 has emerged as a growing global public health challenge requiring patient-centred responses, yet its prevalence and management are often underestimated. We aimed to investigate the rehabilitation status and medical choice behaviours among patients with long COVID-19 in China during the Omicron wave.

**Methods:**

We conducted a national cross-sectional study and a discrete choice experiment (DCE) in China from 4 July to 11 August 2023. We used the modified COVID-19 Yorkshire Rehabilitation Scale (C19-YRSm) to assess rehabilitation status. We collected preferences for medical choice behaviours of long COVID-19, demographics, health-related factors, and COVID-19 history. We assessed preferences for medical choice behaviours among people with long COVID-19 using mixed logit models.

**Results:**

Among 2942 participants health status was significantly poorer than before COVID-19 infection. The prevalence of common symptoms assessed by C19-YRSm ranged from 21.52% to 72.67%, with fatigue (72.67%) being the most common, followed by breathlessness on walking up a flight of stairs (64.96%) and sleep problems (62.95%). Of these symptoms, the majority of participants (15.06–47.65%) reported mild problems. Of the five functional limitations, difficulty with other activities of daily living (31.03%) was the most common, followed by difficulty with communication (28.04%), while difficulty with personal care (9.65%) was the least common. The DCE results showed that the strongest attribute affecting preferences was medical distance (*β* = −1.135). While seeking healthcare for long COVID-19, people preferred lower out-of-pocket costs, a closer distance, a higher hospital level, nutritional supportive therapies or rehabilitation training, and medical services that integrate traditional Chinese and Western medicine.

**Conclusions:**

A substantial proportion of individuals developed long COVID-19 symptoms and functional limitations, most of which were mild. These findings highlight the importance of ongoing screening and comprehensive, tailored rehabilitation services to promote recovery and avert a public health crisis of long COVID-19.

Long COVID-19 (also known as post-COVID-19 condition) is a multisystemic condition following severe acute respiratory syndrome coronavirus 2 (SARS-CoV-2) infection, with more than 200 reported symptoms, affecting physical, cognitive, and mental health domains and lasting for months or years after the initial infection [1–3]. Estimates of long COVID-19 prevalence range from 10% to 80% – this variation is influenced by factors such as differences in study design and the clinical and demographic characteristics of study participants [4]. Based on a conservative estimate of 10% of infected individuals and more than 773 million reported COVID-19 cases globally [5,6], there are at least 77 million individuals worldwide with long COVID-19. With such an overwhelming population, the impacts of long COVID-19 on daily activities, livelihoods, and labour force participation are profound, contributing to labour shortages and economic losses [7–9], which pose a significant and growing burden on health systems, economies, and societies globally [10,11].

Even though advances in diagnosis and treatment for long COVID-19 have been observed recently [8,12,13], many prominent scientific questions remain to be addressed. Firstly, there is an urgent need for systematic, comprehensive screening for long COVID-19 to understand its current burden in countries. In many places, especially in low- and middle-income countries, data on long COVID-19 are absent [9]. The emergence of variants, such as the highly transmissible Omicron, and the increasing prevalence of reinfection might further affect the development of long COVID-19 [2]. Secondly, there is an urgent need for countries to understand the healthcare needs of individuals with long COVID-19 and to develop strategies to provide timely, targeted health services for them. As health systems differ across countries, targeted measures tailored to patients’ demands and preferences are needed to deliver the best healthcare services [14,15], yet research in this field remains limited.

During the Omicron wave, despite its potentially lower risk of long COVID-19 compared to earlier variants, its high transmissibility led to a significant number of cases, and future numbers with long COVID-19 will inevitably rise, making it particularly relevant to assess rehabilitation outcomes in this context [16,17]. From late 2022 to early 2023, China experienced a nationwide epidemic of the Omicron variant, with more than 80% of the population estimated to be infected [18]. As the most populous country in the world, China faces a unique challenge; the sheer scale of COVID-19 survivors will impose substantial long-term medical and socioeconomic burdens [19]. Some studies conducted in China have reported long COVID-19 [20–23], providing important data on the prevalence and disease burden of these symptoms, but these studies mainly focused on patients infected with the wild type or other early variants. Additionally, due to the high heterogeneity in case definitions, assessment tools, follow-up duration, and study population selection across previous studies, the true prevalence of long COVID-19-19 remains unclear [21]. Therefore, there is an urgent need to use a standardised tool to quantitatively assess the rehabilitation status after COVID-19 during the Omicron wave in the Chinese population. The development of tools such as the modified COVID-19 Yorkshire Rehabilitation Scale (C19-YRSm) enables standardised, comprehensive assessments of long COVID-19 [24], thereby enabling effective addressing of this gap.

In addition to understanding the prevalence and rehabilitation status of long COVID-19, it is equally critical to explore patients’ healthcare demands and preferences. Discrete choice experiments (DCEs) are a research method commonly used to study preferences in healthcare [25], which assumes that the characteristics of policies (or projects) can be described with corresponding attributes and levels, and assesses the strength of an individual’s preferences by evaluating the trade-offs between attributes and levels [26]. This method has been widely used in health economics to elicit preferences for healthcare products and programmes, particularly vaccines and medical choice behaviours [27]. In the context of the COVID-19 pandemic, DCEs have been used to study preferences for COVID-19 vaccines [28–31], preventative measures [32–34], restriction policies [35,36], and diagnostic and testing methods [37–39]. The unique nature of long COVID-19, with its wide spectrum of symptoms and healthcare needs, makes it particularly well-suited to a DCE approach. By exploring trade-offs among different healthcare attributes, DCE can capture the nuanced preferences of long COVID-19 patients and identify their priorities for medical services, including accessibility, affordability, and the types of care most valued, which could provide critical insights for designing patient-centred healthcare policies, optimising resource allocation, and addressing the disparities in healthcare access across different regions.

In China, understanding the medical preferences of long COVID-19 patients is particularly urgent, given the country’s vast and diverse healthcare system, which faces significant challenges in accommodating the large number of COVID-19 survivors, especially following the Omicron wave. Therefore, we conducted a nationwide survey in China to investigate the rehabilitation status and preferences for medical choice behaviours among COVID-19 survivors with long COVID-19, using the C19-YRSm and a DCE. We aim to provide valuable references for prevention, management, and rehabilitation strategies of long COVID-19, enabling data-driven resource allocation and the design of patient-centred healthcare policies during the post-pandemic era.

## METHODS

### Study design and participants

To evaluate the rehabilitation status and medical choice behaviour of long COVID-19 among people recovered from SARS-CoV-2 infection during the post-pandemic era, we conducted a national cross-sectional survey with a DCE in China from 4 July to 11 August 2023, nearly six months after the last large-scale nationwide SARS-CoV-2 infections [40]. We distributed anonymous questionnaires *via* Questionnaire Star – a widely used professional data collection platform in China, with more than 6.2 million registered members and nearly 300 million users every month, which can accurately send the electronic questionnaire to our expected representative respondents based on clear personal information (*e.g.* age, gender, and residence) of registered members [41]. Recruitment criteria included agreement to complete the questionnaire carefully, being ≥18 years old, and having a prior SARS-CoV-2 infection. We decided to include participants aged ≥18 years based on ethical considerations, as adult participants can provide informed consent independently. We obtained informed consent, and all respondents consented to the anonymised use of their data for academic purposes.

To determine the sample size, we used the following formula: n = (Z^2^_α/2_ × p(1 − p)) / d^2^

Based on previous studies reporting a prevalence of long COVID-19 of 49% among individuals recovered from SARS-CoV-2 infection in China [22], with a significance level of α = 0.05 and a two-sided confidence interval width of d = 0.05, we estimated the minimum estimated sample size at 1574 . To ensure sufficient and representative participants, we pre-designed a sample size of 3000 participants, which exceeded the minimum requirement. We used quota sampling to allocate the sample proportionally across 31 provinces based on the population distribution reported in the Seventh National Census (Table S1 in the **Online Supplementary Document**). After data collection and cleaning, 2942 participants were eligible and included in the analysis.

### Rehabilitation status

We measured rehabilitation status using the C19-YRSm, which captures the severity of the leading persistent symptoms and functional disability in individuals with long COVID-19 and performs a comprehensive biopsychosocial assessment of a patient’s recovery [24]. The C19-YRSm is a 17-item patient-reported outcome measure rated on a 0–3 numerical rating scale, where zero represents a symptom not present, one a mild problem (*i.e.* not affecting daily life), two a moderate problem (*i.e.* affecting daily life to a certain extent), and three a severe problem (*i.e.* life-disturbing or affecting all aspects of daily life). The C19-YRSm comprises four subscales that assess the severity of patients’ key symptoms, functional limitations, other symptoms, and overall health. It also captures pre-COVID scores for comparison. Items 1–10 form the symptom severity subscale (score 0–30; higher scores indicate worse symptoms), items 11–15 form the functional disability subscale (score 0–15; higher scores indicate worse functional impairment), item 16 forms the other symptoms subscale (score 0–25; higher scores indicate more other symptoms), and item 17 forms the overall health score (score 0–10; higher scores indicate better overall health status).

Compared to the measures used in the previous long COVID-19 study, C19-YRSm is advantageous because it is comprehensive, covering most symptoms, less burdensome, and condition-specific [24]. Additionally, the World Health Organization’s International Classification of Functioning, Disability and Health provide a framework for understanding the relationship between different aspects of any health condition. The domains covered by the C19‐YRSm, when mapped to the International Classification of Functioning, Disability and Health components, show that there is satisfactory capture of all the components (*i.e.* body functions and structures, activities, participation, environmental factors, and personal factors), making it suitable for a comprehensive biopsychosocial assessment of the condition [24]. The C19-YRSm has been translated into Chinese and cross-culturally adapted and has demonstrated good reliability and validity in China [42].

### DCE

We adhered to the International Society for Pharmacoeconomics and Outcomes Research reporting guidelines for the design and reporting of the research question, survey, tasks, instrument, and analyses [43]. We conducted a rapid review of articles extracted from PubMed, Embase, Scopus, Web of Science, WANFANG DATA, and CNKI from their inception until 1 June 2023, to form an attributes pool of factors affecting decision-making regarding medical choice behaviours of long COVID-19. After conducting a focus group discussion with experts to rank the shortlisted attributes and to identify which attributes were potentially significant, we ultimately identified five attributes, each with three levels: hospital level (primary, secondary, and tertiary hospital), medical distance (20, 40, and 60 minutes), out-of-pocket costs per visit (100, 200, and 300 CNY), therapeutic method (medication-based symptom relief, rehabilitation training, and nutrition support), and medical service type (traditional Chinese, integrated traditional Chinese and Western, and Western medicine). Usually, a full factorial design that combines all possible combinations is considered ideal. However, we could not use it, as it would generate 3^5^ × (3^5^ − 1) = 58 806 choice sets, imposing a high cognitive burden on respondents. Therefore, we used an orthogonal design to identify 18 combinations, which we then randomised in pairs to form eight choice sets. Finally, each DCE task comprised nine choice sets, each containing two alternatives [44]. Participants were asked to choose between alternatives, assuming they remain uncomfortable for a while after COVID-19 infection and decide to seek medical care due to long COVID-19 symptoms (Figure S1 in the **Online Supplementary Document**).

### Covariates

In addition to rehabilitation status and medical choice behaviours of long COVID-19 symptoms, we also collected sociodemographic characteristics (*i.e.* gender, age, location, education level, relationship status, and income level), health-related factors (*i.e.* smoking status, drinking status, chronic disease history), COVID-19 vaccination status, and COVID-19 infection history (*i.e.* number, time and severity of SARS-CoV-2 infection).

### Statistics analysis

We calculated the prevalence of long COVID-19 symptoms and scores for items and sub-scales assessed by the C19-YRSm. We assessed the normality of continuous variables by examining histograms and Q-Q plots of residuals. We presented normally distributed variables as mean (x̄) (standard deviation (SD)) and non-normally distributed variables as median (interquartile range (IQR)). We used frequencies and percentages to describe categorical variables. We used t-tests or analysis of variance to compare group differences in C19-YRSm scale scores, a paired t-test to compare scores before and after COVID-19 infection, and Spearman’s rank correlation to examine the relationships among the subscales. In addition, we performed subgroup analyses based on participants’ gender, age, number of COVID-19 infections, and the time of the most recent infection.

We used a mixed logit model to assess respondents’ preferences for medical choice behaviours regarding long COVID-19 symptoms [45]. We applied dummy coding to four non-linear attributes, except out-of-pocket costs per visit, which was defined as a continuous numeric variable in the model. We estimated the utility component (*i.e.* V), which describes the measurable utility of a specific healthcare based on the attributes that were included in the DCE, using the following model:

V = *β*_0_ + *β*_1_ × out-of-pocket costs per visit + *β*_2_ × hospital level_tertiary hospital_ + *β*_3_ × medical distance_60 min_ + *β*_4_ therapeutic method_rehabilitation training_ + *β*_5_ × therapeutic method_nutrition support_ + *β*_6_ × medical service type_integrated traditional Chinese and Western medicine_ + *β*_7_ × medical service type_Western medicine_

where *β*_0_ represents the alternative-specific constant, and *β*_1–7_ are the attribute-level estimates indicating the relative importance of each attribute level.

We used Stata, version 16.0 (StataCorp LLC, College Station, Texas, USA) for the mixed logit model and SPSS, version 29.0 (IBM Corp., Armonk, New York, USA) for all other analyses. We considered a two-sided *P*-value of 0.05 to be statistically significant.

## RESULTS

### Characteristics of participants

Among the 2942 participants included in the analysis (**Table 1**), 1757 (59.72%) were female, 1390 (47.25%) were <30 years old, 2662 (90.48%) lived in the urban areas, 1928 (65.53%) had at least a Bachelor’s degree, and 1954 (66.42%) had no chronic disease. Only 519 (17.64%) participants have been infected with SARS-CoV-2 ≥2 times. Among 2942 participants with the most recent SARS-CoV-2 infection, most (75.80%) had mild symptoms, and only 0.48% had severe or critical infection. Around a third (32.90%) of the participants were recently infected with SARS-CoV-2 in the past six months, 58.94% in the past 7–12 months, and 8.16% in the past >12 months.

**Table 1:** Characteristics of participants and scores for symptom severity, functional ability, other symptoms, and overall health assessed by the C19-YRSm*

		Symptom severity		Functional ability		Other symptoms		Overall health	
	**Total, n (%)**	**x̄ (SD)**	***P*-value**	**x̄ (SD)**	***P*-value**	**x̄ (SD)**	***P*-value**	**x̄ (SD)**	***P*-value**
**Total**	2942 (100.00)	8.63 (6.21)		1.47 (2.15)		1.76 (1.69)		6.9 (2.16)	
**Gender**			0.002		0.740		0.001		0.002
Male	1185 (40.28)	8.19 (6.29)		1.45 (2.16)		1.63 (1.65)		8.04 (2.12)	
Female	1757 (59.72)	8.92 (6.14)		1.48 (2.15		1.85 (1.72)		7.8 (2.17)	
**Age in years**			0.008		0.026		0.022		0.526
<30	1390 (47.25)	8.91 (6.22)		1.49 (2.11)		1.80 (1.68)		6.86 (2.18)	
30–34	790 (26.85)	8.77 (6.01)		1.48 (2.12)		1.83 (1.69)		6.99 (2.01)	
35–39	421 (14.31)	8.02 (6.20)		1.21 (2.00)		1.69 (1.75)		6.86 (2.10)	
≥40	341 (11.59)	7.91 (6.58)		1.67 (2.50)		1.52 (1.64)		6.89 (2.43)	
**Location**			0.119		0.030		0.301		0.181
Urban	2662 (90.48)	8.57 (6.18)		1.44 (2.12)		1.75 (1.68)		7.92 (2.14)	
Rural	280 (9.52)	9.2 (6.44)		1.73 (2.38)		1.86 (1.77)		7.73 (2.28)	
**Education**			0.028		<0.001		0.678		0.194
High school and below	765 (26.00)	8.89 (6.21)		1.59 (2.23)		1.79 (1.73)		6.79 (2.33)	
Bachelor’s degree	1928 (65.53)	8.42 (6.12)		1.36 (2.08)		1.74 (1.65)		6.95 (2.07)	
Master’s degree	249 (8.46)	9.39 (6.83)		1.89 (2.36)		1.82 (1.89)		6.82 (2.27)	
**Relationship status**			0.047		0.114		0.058		0.604
Without partner	627 (21.31)	8.2 (6.05)		1.35 (1.98)		1.64 (1.74)		7.86 (2.35)	
With partner	2315 (78.69)	8.74 (6.25)		1.5 (2.19)		1.79 (1.68)		7.91 (2.10)	
**Income level**			0.030		0.013		0.362		0.008
1	39 (1.33)	10.33 (7.22)		2.51 (3.02)		2.03 (1.87)		6.03 (2.61)	
2	210 (7.14)	9.11 (6.36)		1.61 (2.39)		1.69 (1.74)		6.68 (2.32)	
3	714 (24.27)	8.75 (6.27)		1.52 (2.17)		1.7 (1.58)		6.87 (2.19)	
4	1303 (44.29)	8.73 (6.12)		1.44 (2.06)		1.82 (1.75)		6.88 (2.09)	
5	676 (22.98)	8.05 (6.20)		1.35 (2.15)		1.71 (1.67)		7.08 (2.14)	
**Smoking status**			<0.001		<0.001		0.154		0.871
Never/former	2493 (84.74)	8.45 (6.07)		1.39 (2.06)		1.74 (1.69)		7.89 (2.15)	
Current	449 (15.26)	9.61 (6.84)		1.9 (2.54)		1.87 (1.71)		7.91 (2.19)	
**Drinking status**			0.003		0.011		0.020		0.182
Never/former	1660 (56.42)	8.33 (6.13)		1.38 (2.10)		1.7 (1.68)		7.94 (2.20)	
Current	1282 (43.58)	9.01 (6.29)		1.58 (2.22)		1.84 (1.71)		7.84 (2.10)	
**Chronic disease**			<0.001		<0.001		<0.001		<0.001
No	1954 (66.42)	7.72 (6.00)		1.22 (1.97)		1.53 (1.60)		8.01 (2.25)	
Yes	988 (33.58)	10.41 (6.23)		1.96 (2.40)		2.22 (1.77)		7.67 (1.94)	
**COVID-19 vaccination status**			0.443		0.033		0.238		0.022
Unvaccinated or partially vaccinated	266 (9.04)	8.46 (6.27)		1.32 (1.99)		1.82 (1.79)		6.65 (2.35)	
Full vaccination	1127 (38.31)	8.73 (6.23)		1.47 (2.12)		1.81 (1.67)		6.88 (2.11)	
1 dose of booster vaccination	721 (24.51)	8.33 (5.93)		1.33 (2.08)		1.65 (1.66)		7.09 (1.95)	
≥2 doses of booster vaccination	828 (28.14)	8.79 (6.41)		1.63 (2.29)		1.78 (1.71)		6.84 (2.31)	
**SARS-CoV-2 infection history**			0.031		0.809		0.113		0.158
1 time	2423 (82.36)	8.51 (6.23)		1.46 (2.15)		1.74 (1.71)		7.92 (2.17)	
≥2 times	519 (17.64)	9.15 (6.11)		1.49 (2.16)		1.87 (1.60)		7.78 (2.06)	
**Severity of the most recent infection**			<0.001		0.002		<0.001		0.007
Asymptomatic	151 (5.13)	8.15 (7.54)		1.75 (2.50)		1.44 (1.61)		6.96 (2.73)	
Mild	2230 (75.80)	8.27 (6.00)		1.38 (2.10)		1.67 (1.66)		6.96 (2.11)	
Moderate	547 (18.59)	10.13 (6.42)		1.74 (2.24)		2.21 (1.79)		6.65 (2.14)	
Severe/critical	14 (0.48)	11.86 (5.43)		1.57 (2.38)		2.36 (1.55)		5.86 (1.92)	
**Time of the most recent infection, in months**			<0.001		<0.001		0.021		0.541
≤6	968 (32.90)	8.89 (6.08)		1.46 (2.11)		1.84 (1.76)		6.89 (2.05)	
7–12	1734 (58.94)	8.29 (6.13)		1.39 (2.10)		1.69 (1.64)		6.92 (2.19)	
>12	240 (8.16)	9.97 (7.04)		2.02 (2.58)		1.93 (1.81)		6.76 (2.33)	

C19-YRSm – Modified COVID-19 Yorkshire Rehabilitation Scale, SD – standard deviation, x̄ – mean

*‘Location’ included urban areas (*i.e.* main urban areas, urban-rural junction, and peri-urban areas) and rural areas (*i.e.* townships and villages); ‘high school and below’ included high school and below, technical secondary school, junior college, and undergraduate students; master candidates were also included in the ‘Master’s degree’. For ‘relationship status’, we divided participants into two categories based on whether they had a lover or a cohabiting spouse, with married but separated considered unpartnered. ‘Income level’ represents after-tax monthly household income *per capita*, labelled as 1 (0–999 CNY), 2 (1000–2999 CNY), 3 (3000–4999 CNY), 4 (5000–9999 CNY), and 5 (≥10 000 CNY).

Regarding rehabilitation status assessed by the C19-YRSm (**Table 1**), the x̄ score for symptom severity was 8.63 (SD = 6.21), 1.47 (SD = 2.15) for functional ability, 1.76 (SD = 1.69) for other symptoms, and 6.9 (SD = 2.16) for overall health. Participants who were female, lived in rural areas, currently drank or smoked, had a chronic disease history, had been infected with SARS-CoV-2 ≥2 times, and were recently infected with SARS-CoV-2 in the past >12 months showed higher impairment across categories and worse overall health after SARS-CoV-2 infection (*P* < 0.05).

### Prevalence of common symptoms, functional limitations, and other symptoms captured by the C19-YRSm

**Figure 1:**
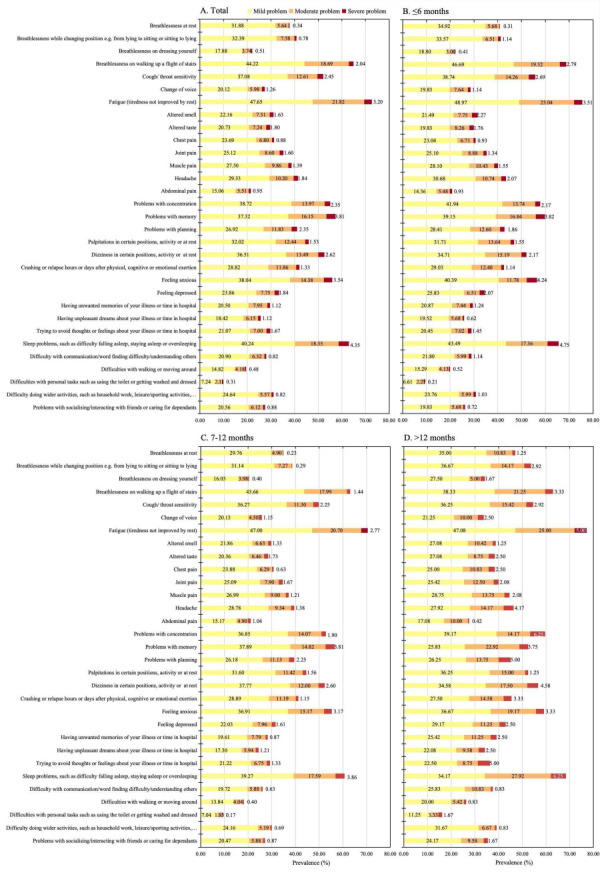
Prevalence of common symptoms and functional limitations after COVID-19 infection assessed by the C19-YRSm, by the most current COVID-19 infection time. **Panel A.** Total. **Panel B.** ≤6 months. **Panel C.** 7–12 months. **Panel D.** >12 months. C19-YRSm – Modified COVID-19 Yorkshire Rehabilitation Scale

The prevalence of common symptoms assessed by the C19-YRSm ranged from 21.52% to 72.67% (**Figure 1**, Panels A–D; **Table 2**), the most common symptom was fatigue (72.67%), followed by breathlessness on walking up a flight of stairs (64.96%) and sleep problems such as difficulty falling asleep, staying asleep or oversleeping (62.95%). Of these symptoms, the majority of participants (15.06–47.65%) reported mild problems that did not affect their daily lives. Only a few participants (0.34–4.35%) reported severe problems affecting all aspects of daily life. Of the five functional limitations, difficulty with other activities of daily living, such as household work, leisure/sporting activities, paid/unpaid work, study, or shopping (31.03%), was the most common, followed by difficulty with communication/word-finding difficulty/understanding others (28.04%). Difficulty with personal care, such as using the toilet or getting washed and dressed (9.65%), was the least common. Subgroup analyses showed that the prevalence of most of these symptoms was highest among those infected for >12 months, followed by those infected within 7–12 months (**Figure 1**, Panels C and D; Table S2 in the **Online Supplementary Document**). The prevalence of most of these symptoms was higher among participants who had been infected with SARS-CoV-2 ≥2 times than those infected only once (Table S3 in the **Online Supplementary Document**) and was also higher among women than men (Table S4 in the **Online Supplementary Document**).

**Table 2:** Prevalence of common symptoms and severity scores before and after COVID-19 infection assessed by the C19-YRSm

	Prevalence, n (%)	Current score*	Pre-COVID score*	∆ score*	*P*-value
**Symptom severity**		8.63 (6.21)	5.05 (5.26)	3.58 (6.17)	<0.001
Breathlessness		1.03 (0.78)	0.63 (0.72)	0.40 (0.81)	<0.001
At rest	1114 (37.87)	0.44 (0.62)	0.21 (0.49)	0.23 (0.66)	<0.001
Changing position *e.g.* from lying to sitting or sitting to lying	1199 (40.75)	0.50 (0.67)	0.26 (0.55)	0.24 (0.70)	<0.001
On dressing yourself	651 (22.13)	0.27 (0.55)	0.17 (0.46)	0.10 (0.56)	<0.001
On walking up a flight of stairs	1911 (64.96)	0.88 (0.78)	0.49 (0.65)	0.39 (0.82)	<0.001
Cough/throat sensitivity/voice change		0.77 (0.82)	0.42 (0.70)	0.36 (0.95)	<0.001
Cough/throat sensitivity	1534 (52.14)	0.70 (0.78)	0.36 (0.65)	0.34 (0.95)	<0.001
Change of voice	805 (27.36)	0.36 (0.65)	0.21 (0.53)	0.15 (0.71)	<0.001
Fatigue	2138 (72.67)	1.01 (0.79)	0.54 (0.69)	0.47 (0.88)	<0.001
Smell/taste		0.54 (0.79)	0.28 (0.63)	0.26 (0.86)	<0.001
Altered smell	915 (31.10)	0.42 (0.70)	0.21 (0.54)	0.21 (0.79)	<0.001
Altered taste	876 (29.78)	0.41 (0.70)	0.21 (0.55)	0.20 (0.78)	<0.001
Pain/discomfort		0.92 (0.86)	0.56 (0.78)	0.36 (0.94)	<0.001
Chest pain	923 (31.37)	0.40 (0.66)	0.20 (0.51)	0.20 (0.69)	<0.001
Joint pain	1039 (35.32)	0.47 (0.72)	0.27 (0.57)	0.21 (0.75)	<0.001
Muscle pain	1140 (38.75)	0.51 (0.73)	0.27 (0.59)	0.25 (0.81)	<0.001
Headache	1217 (41.37)	0.55 (0.75)	0.33 (0.63)	0.23 (0.85)	<0.001
Abdominal pain	633 (21.52)	0.29 (0.61)	0.17 (0.48)	0.12 (0.58)	<0.001
Cognition		1.05 (0.88)	0.64 (0.77)	0.41 (0.82)	<0.001
Problems with concentration	1619 (55.03)	0.74 (0.78)	0.41 (0.63)	0.33 (0.75)	<0.001
Problems with memory	1685 (57.27)	0.81 (0.84)	0.40 (0.64)	0.41 (0.79)	<0.001
Problems with planning	1209 (41.09)	0.58 (0.79)	0.36 (0.65)	0.21 (0.66)	<0.001
Palpitations/dizziness		0.85 (0.84)	0.50 (0.69)	0.35 (0.80)	<0.001
Palpitations	1353 (45.99)	0.61 (0.76)	0.32 (0.59)	0.30 (0.73)	<0.001
Dizziness	1548 (52.62)	0.71 (0.80)	0.40 (0.64)	0.31 (0.77)	<0.001
Post-exertional malaise (worsening of symptoms)	1236 (42.01)	0.57 (0.75)	0.29 (0.57)	0.28 (0.70)	<0.001
Anxiety/mood		1.00 (0.88)	0.65 (0.77)	0.35 (0.76)	<0.001
Feeling anxious	1646 (55.95)	0.77 (0.82)	0.46 (0.66)	0.31 (0.75)	<0.001
Feeling depressed	984 (33.45)	0.45 (0.72)	0.28 (0.58)	0.17 (0.61)	<0.001
Having unwanted memories of your illness or time in hospital	870 (29.57)	0.40 (0.68)	0.23 (0.53)	0.17 (0.63)	<0.001
Having unpleasant dreams about your illness or time in hospital	756 (25.70)	0.34 (0.64)	0.19 (0.49)	0.15 (0.59)	<0.001
Trying to avoid thoughts or feelings about your illness or time in hospital	875 (29.74)	0.40 (0.69)	0.24 (0.56)	0.16 (0.63)	<0.001
Sleep problems	1852 (62.95)	0.90 (0.85)	0.54 (0.70)	0.36 (0.81)	<0.001
**Functional ability**		1.47 (2.15)	0.86 (1.82)	0.61 (2.05)	<0.001
Difficulty with communication	825 (28.04)	0.36 (0.64)	0.20 (0.49)	0.16 (0.56)	<0.001
Difficulties with walking or moving around	573 (19.48)	0.25 (0.55)	0.15 (0.45)	0.09 (0.58)	<0.001
Difficulties with personal care	284 (9.65)	0.12 (0.41)	0.09 (0.37)	0.03 (0.42)	<0.001
Difficulty with other activities of daily living	913 (31.03)	0.38 (0.63)	0.20 (0.51)	0.18 (0.66)	<0.001
Problems with social role	811 (27.57)	0.35 (0.64)	0.21 (0.52)	0.14 (0.63)	<0.001
**Other symptoms**		1.76 (1.69)			
**Overall health**		6.90 (2.16)	7.31 (2.47)	−0.42 (1.96)	<0.001

C19-YRSm – Modified COVID-19 Yorkshire Rehabilitation Scale, ∆ – delta

*Values are presented as mean (standard deviation).

**Figure 2:**
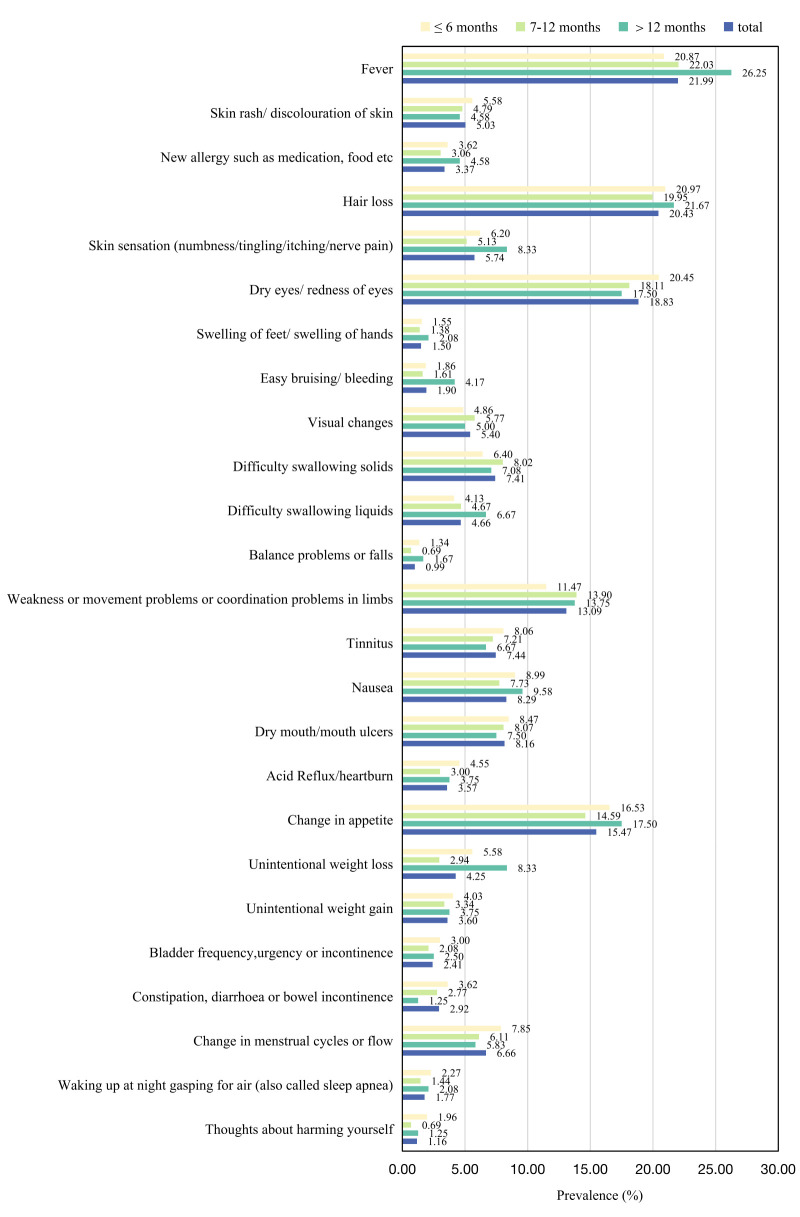
Prevalence of other symptoms after COVID-19 infection assessed by the C19-YRSm, by the most current COVID-19 infection time. C19-YRSm – Modified COVID-19 Yorkshire Rehabilitation Scale

As for the other 25 symptoms captured by the C19-YRSm (**Figure 2**), the most common were fever (21.99%), hair loss (20.43%), dry eyes/redness of eyes (18.83%), and change in appetite (15.47%). The prevalence of other symptoms varied among participants with different characteristics (Table S6–9 in the **Online Supplementary Document**). Hair loss was the most common other symptom among participants who were infected within the last six months (20.97%), females (24.76%), and those aged 35–39 years (19.24%). Dry eyes/redness of eyes was the most common other symptom among participants who were infected ≥2 times (22.16%), aged 30–34 years (20.76%), and aged ≥40 years (17.01%).

### Severity of common symptoms and functional limitations

**Figure 3:**
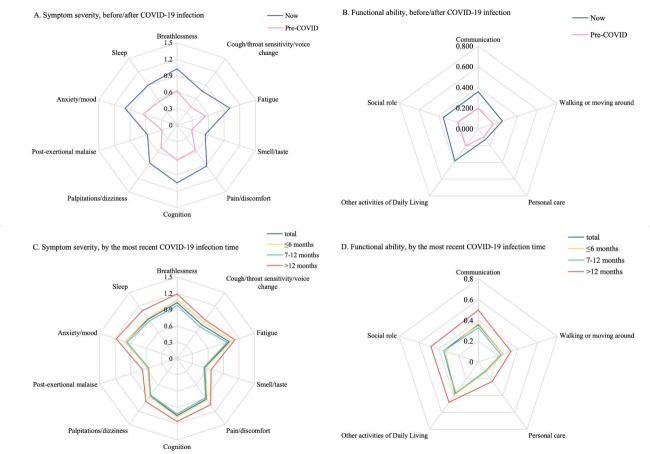
Radar plot of the mean severity of ten common symptoms and five functional abilities assessed by the C19-YRSm. **Panel A.** Symptom severity, before/after COVID-19 infection. **Panel B.** Functional ability, before/after COVID-19 infection. **Panel C**. Symptom severity, by the most recent COVID-19 infection time. **Panel D**. Functional ability, by the most recent COVID-19 infection time. C19-YRSm – Modified COVID-19 Yorkshire Rehabilitation Scale

All common symptoms and functional limitations were more severe now than pre-COVID (**Figure 3**, Panels A and B; Table S10 in the **Online Supplementary Document**). The most severe common symptom was cognition problems, such as problems with concentration, memory or planning (x̄ = 1.05), indicating that these problems were moderate and might affect daily life to some extent. Regarding the five functional abilities, the most severe was difficulty doing other activities of daily living, such as household work, leisure/sporting activities, paid/unpaid work, study or shopping (x̄ = 0.38). However, according to the scale scoring criteria, this functional limitation was mild and would not affect daily life.

Subgroup analyses showed that common symptoms and functional limitations were more severe among participants with the most recent SARS-CoV-2 infection>12 months ago, females, and those infected ≥2 times (**Figure 3**, Panels C and D; Table S11–13 in the **Online Supplementary Document**). Additionally, participants aged ≥30 years showed greater severity in most common symptoms and functional abilities assessed by the C19-YRSm (Table S14 in the **Online Supplementary Document**).

### Correlation between subscales of C19-YRSm

There was a significant positive correlation between scores for common symptoms, functional ability, and other symptoms (*P* < 0.001) (Figure S2 in the **Online Supplementary Document**). In contrast, overall health was negatively correlated with scores for symptom severity, functional ability, and other symptoms (*P* < 0.001) (Table S15 in the **Online Supplementary Document**).

### Preference for medical choice behaviour of long COVID-19

The most important attribute level for participants who recovered from COVID-19 was the medical distance of 60 minutes (*β* = −1.135; *P* < 0.001), indicating that participants did not prefer a greater distance from medical services when seeking healthcare due to post-COVID-19 symptoms (**Table 3**). Western medicine (*β* = −0.680; *P* < 0.001) also showed a negative effect, suggesting that people were less attracted to Western medicine than traditional Chinese medicine. The effect of the ‘out-of-pocket costs per visit’ attribute on medical service-seeking behaviour (*β* = −0.627; *P* < 0.001) was also negative, suggesting that people tended to choose medical behaviours with lower costs. The coefficients of attribute levels including tertiary hospital (*β* = 0.294; *P* < 0.001), rehabilitation training (*β* = 0.213; *P* < 0.001), nutrition support (*β* = 0.255; *P* < 0.001), and integrated traditional Chinese and Western medicine (*β* = 0.238; *P* < 0.001) were positive and statistically significant, suggesting that people were more likely to choose programmes that included these attribute levels when seeking medical service because of long COVID-19 symptoms when other factors were equal.

**Table 3:** Main effect analysis results of mixed logit model

	*β*	SE	*P*-value
**Out-of-pocket costs per visit**	−0.627	0.018	<0.001
**Hospital level**			
Primary hospital	ref		
Tertiary hospital	0.294	0.027	<0.001
**Medical distance, in minutes**			
20	ref		
60	−1.135	0.033	<0.001
**Therapeutic method**			
Medication-based symptom relief	ref		
Rehabilitation training	0.213	0.038	<0.001
Nutrition support	0.255	0.041	<0.001
**Medical service type**			
Traditional Chinese medicine	ref		
Integrated traditional Chinese and Western medicine	0.238	0.036	<0.001
Western medicine	−0.680	0.045	<0.001
**ASC**	−0.112	0.017	<0.001
**Model fit statistics**			
Number of observations	52 956		
Wald χ^2^ (df = 7)	2349.630		<0.001
Log simulated likelihood	−14 801.635		

ASC – alternative specific constant, df – degrees of freedom, SE – standard error, ref – reference, χ^2^ – chi-square

Subgroup analyses showed similar results across participants in different age groups, those infected with SARS-CoV-2 only once, and those whose most recent infection was within the past nine months (Table S16–18 in the **Online Supplementary Document**). However, the effect of ‘integrated traditional Chinese and Western medicine’ was not significant among participants infected ≥2 times (*P =* 0.126). Among participants who were recently infected in the past ≥10 months, the effects of ‘tertiary hospital’, ‘nutrition support’, and ‘integrated traditional Chinese and Western medicine’ were not significant (*P >* 0.05). Lastly, the effect of ‘rehabilitation training’ also became insignificant in participants whose last SARS-CoV-2 infection was more than 12 months ago (*P =* 0.185).

## DISCUSSION

To our knowledge, this is the first nationwide survey of rehabilitation status six months after the most recent Omicron variant outbreak in China, and by embedding a DCE, we not only comprehensively assessed the rehabilitation status of COVID-19 infection survivors but also explored their preferences for medical consultations of long COVID-19. Of 2942 participants, all were in poorer health than before their infections. The prevalence of common symptoms assessed by the C19-YRSm ranged from 21.52% to 72.67%, with fatigue, breathlessness on walking up a flight of stairs, and sleep problems being particularly common. Of these symptoms, most participants reported mild problems. While seeking healthcare for long COVID-19, people preferred lower out-of-pocket costs, a closer distance, a higher hospital level, nutritional supportive therapies or rehabilitation training, and medical services that integrate traditional Chinese and Western medicine. These findings underscore the need for ongoing screening, comprehensive rehabilitation, and tailored healthcare services to support recovery from long COVID-19. They also provide guidance to governments and healthcare organisations on delivering timely, patient-centred care in the post-pandemic era.

We found disparities in the rehabilitation status after COVID-19 infection among respondents with different characteristics. Females, participants who lived in rural areas, currently drank or smoked, had a chronic disease history, were infected with SARS-CoV-2 ≥2 times, and were infected with SARS-CoV-2 in the past >12 months, were in poorer rehabilitation status. Women are considered a high-risk group for long COVID-19. Studies have suggested that the gender-specific pattern of long COVID-19 symptoms differs from that of severe acute COVID-19, which tends to affect men more severely [46]. This difference indicates that the underlying pathological mechanisms of long COVID-19 may diverge from those of acute infection [46]. Generally, women experience less severe disease from viral infections, have stronger antibody responses, and exhibit higher rates of adverse reactions to vaccines and antiviral drugs [46,47]. X chromosome-linked genes are believed to be associated with susceptibility to viral infections and autoimmune diseases, supporting the role of autoimmunity in the pathogenesis of long COVID-19 [46,47]. Previous studies suggested that socioeconomic factors were fundamental determinants of health outcomes following COVID-19 [48]. Individuals from low- and middle-income countries, marginalised communities, and socially disadvantaged groups might experience more severe impacts [48]. Rural residents might face barriers such as limited access to healthcare services, fewer specialised medical resources, and longer travel distances to medical facilities [49], which may delay timely diagnosis and treatment of long COVID-19. Additionally, rural areas may have lower health literacy and fewer rehabilitation programmes [50], exacerbating disparities in recovery outcomes. The realisation of health equity requires that patients of all genders and from diverse residential areas can access appropriate health services in a timely and equitable manner [51]. While diagnosing COVID-19 infection, clinicians should pay closer attention to these susceptible and vulnerable groups to enable subsequent long-term evaluation for timely and effective rehabilitation and treatment of long COVID-19. Moreover, C19-YRSm could also be widely used as a screening tool for long COVID-19 and as an assessment tool for rehabilitation status in populations, as it is easily understood and manipulated [24].

We found that the most common long COVID-19 symptom was fatigue (72.67%), though most reported mild symptoms. Results of a cohort study conducted in China also showed that fatigue or muscle weakness was the most common symptom at six months (52%), 12 months (20%), and two years (30%) of follow-up after discharge from the hospital, regardless of disease severity [20–22]. A meta-analysis across multiple countries also showed that fatigue was the most reported of more than 50 long COVID-19 symptoms [52]. Previous studies showed that fatigue was also the most common symptom among SARS survivors and could last up to four years [53]. The cause and pathogenesis of fatigue after COVID-19 are still unclear. However, based on previous evidence from SARS, potential factors that might have played a role in this condition included impairment of lung diffusion capacity and extrapulmonary causes, such as viral-induced myositis during the early stages of the illness, disturbance in cytokine levels, muscle wasting and deconditioning, or the possibility of corticosteroid-induced myopathy [22,53–57]. It is also possible that a combination of these factors could have contributed to the observed symptoms [22].

Although most participants reported mild problems that did not affect their daily lives, the high prevalence of common symptoms should not be overlooked. These symptoms might also affect individuals’ daily functioning, quality of life, and work productivity. In Canada, more than one-fifth of adults with long COVID-19 reported that their daily lives were limited due to long COVID-19 [58]. Previous studies have shown that persistent symptoms following COVID-19 might impact patients’ ability to work and employment status, individuals with long COVID-19 were less likely to engage in full-time work (odds ratio = 0.84; 95% confidence interval = 0.74–0.96) and faced a higher risk of unemployment (odds ratio = 1.23; 95% confidence interval = 1.02–1.48) [59]. A longitudinal cohort study conducted among early COVID-19 survivors hospitalised in Wuhan, China, found that 11% of patients had not returned to work two years after discharge [21]. These findings highlight the critical need for tailored interventions to mitigate fatigue and other symptoms and improve rehabilitation outcomes. Therefore, there is a need for increased awareness and education regarding long COVID-19 and rehabilitation. Healthcare systems should be equipped to provide a range of medical services to meet individual needs related to long COVID-19. According to current guidelines and care models [60,61], online self-management support is recommended as a first-line intervention for managing long COVID-19, prior to health service use, and is also widely referenced in the management of long COVID-19 in China.

Results of the DCE showed that, when seeking healthcare for long COVID-19, people preferred lower out-of-pocket costs, a shorter distance, and a higher hospital level, consistent with previous studies [62]. In China, public hospitals are divided into three grades based on their functions, facilities, and technical capabilities, with tertiary hospitals at the highest level. The apparent preference for tertiary hospitals over primary hospitals may be due to the unequal distribution of medical resources [62]. Although the Chinese government has introduced medical alliances within a hierarchical healthcare system to optimise resource allocation and improve services in underdeveloped areas [63], a preference for tertiary hospitals for long COVID-19 care may strain resources and limit access for patients with other conditions.

Participants in this study were more likely to choose nutritional supportive therapies or rehabilitation training to relieve long COVID-19 symptoms. Effective rehabilitation interventions for long COVID-19 are critical. Previous reviews showed that rehabilitation interventions for patients with long COVID-19 encompassed a range of approaches, including respiratory exercises, aerobic training, strength exercises, and medication treatment, among which rehabilitation training was the most common and effective for improving functional capacity and quality of life [64–66]. Additionally, Tosato and colleagues suggested that bioactive foods, supplements, and nutraceuticals could be used to manage long-term COVID-19 clinical sequelae [67]. As there is no definitive treatment for COVID-19 during the recovery period, clinical management primarily relies on symptomatic treatment [68]. Although medication interventions play a role in the management of long COVID-19, participants in our study did not prefer medication-based symptom-relief therapy, possibly because medications do not eliminate the problem at its root and concerns about possible side effects.

We found that participants preferred traditional Chinese medicine or a combination with Western medicine. Traditional Chinese medicine has a long history and cultural relevance in China, where it is widely perceived as a holistic and natural approach to health [69]. Previous studies showed that traditional Chinese medicine was effective for treating COVID-19 during the recovery period, effectively alleviating clinical symptoms, regulating immunity, and improving pulmonary fibrosis [68]. These findings might strengthen the public trust and reliance on traditional Chinese medicine for managing long COVID-19. The accessibility of traditional Chinese medicine in China’s healthcare system further supports its widespread use. The Chinese government has long supported the development and integration of traditional Chinese medicine into healthcare. In 2019, the State Council issued the ‘Opinions on Promoting the Inheritance and Innovation of Traditional Chinese Medicine’, emphasising the equal importance of traditional Chinese medicine and modern medicine, enhancing traditional Chinese medicine services, and fostering coordinated development [70]. Many public hospitals have integrated traditional Chinese medicine services, making it a practical and affordable option for patients. These findings also underscore the importance of integrating traditional Chinese medicine and other integrative approaches into long COVID-19 management.

In this study, we systematically assessed the rehabilitation status and medical choice behaviour of long COVID-19 nearly six months after the most recent nationwide Omicron epidemic in China in late 2022. Our results are representative of a quota-random sampling method across provinces. We used the C19-YRSm to provide a quantitative, accurate, and comprehensive assessment of the rehabilitation status of COVID-19 survivors. Additionally, the inclusion of a DCE is highly innovative, marking the first application of this method to explore patients recovered from COVID-19 infection's long-COVID-related healthcare behavioural preferences in China. These findings could offer valuable insights for optimising healthcare resource allocation in the post-pandemic era.

However, this study also has some limitations. Firstly, as this was a cross-sectional study, we could not estimate the incidence of long COVID-19 symptoms or make causal inferences. Additionally, as we relied on an online survey, individuals without reliable internet access – particularly those in remote or underserved areas – may have been underrepresented, potentially introducing selection bias. Thirdly, as we asked the respondents to recall their pre-infection symptom severity and functional status, the findings may be subject to recall bias. Furthermore, while we focused on adults aged ≥18 years, it is important to acknowledge that younger individuals, including adolescents and children, can also experience long COVID-19 [50]. Excluding this population limits the generalisability of our findings to younger age groups in China. Future studies are needed to explore the rehabilitation of long COVID-19 among younger individuals to provide more insights. Moreover, after data cleaning, we included 2942 eligible participants, which was below the pre-designed sample size of 3000. However, this sample size still exceeds the minimum estimated requirement of 1574 participants and is sufficient to ensure the representativeness and robustness of our findings. Finally, long COVID-19 is a fluctuating or episodic disease, and the symptom severity might change over time in the same individual. Therefore, it is recommended that patients recovered from COVID-19 be monitored longitudinally using the C19-YRSm to capture fluctuating long COVID-19 symptoms and rehabilitation status [24].

## CONCLUSIONS

We provide valuable references for the rehabilitation status and preference for medical choice behaviour of long COVID-19 among COVID-19 survivors in China during the Omicron wave. Our results showed that nearly seven out of ten individuals developed long COVID-19 symptoms and functional limitations after recovering from acute COVID-19. However, most of them were mild. These findings highlight the importance of ongoing screening, comprehensive rehabilitation support, and tailored healthcare services. For policymakers, it is important to prioritise funding for rehabilitation programmes and enhance public education on long COVID-19. Additionally, efforts should be made to improve access to healthcare services, particularly in rural areas, and to integrate traditional Chinese medicine into rehabilitation programmes to promote recovery and avert a public health crisis caused by long COVID-19 during the post-pandemic era.

## Additional material


Online Supplementary Document
