# Supplementary material for: Rehabilitation status and preference for medical choice behaviours of long COVID-19 in China: a national cross-sectional study and discrete choice experiment
Source: J Glob Health. 2026 Mar 20;16:04081. doi: 10.7189/jogh.16.04081 (PMC13002172; doi:10.7189/jogh.16.04081)
Supplement: Online Supplementary Document [file jogh-16-04081-s001.pdf]

Supplement to: Deng J, Tao L, Liu N, Li J, Qin C, Yan W, Wang Y, Du M, Liu Q, Liu J. Rehabilitation status and preference for medical choice behaviours of long COVID-19 in China: a national cross-sectional study and discrete choice experiment. J Glob Health. 2026;16:04081.

Supplementary Document

Rehabilitation Status and Preference for Medical Choice Behaviors of Long COVID in China: A National Cross-sectional Study and Discrete Choice Experiment

Authors:

Jie Deng, Liyuan Tao, Nan Liu, Jun Li, Chenyuan Qin, Wenxin Yan, Yaping Wang, Min Du, Qiao Liu, Jue Liu#

#Corresponding author

Prof. Jue Liu, Department of Epidemiology and Biostatistics, School of Public Health, Peking University, Beijing 100191, China.
Tel: 86-10-8 2801528-316, Fax: 86-10-8 2805146, Email: jueliu@bjmu.edu.cn

Content

Table S1. Sample proportion and sample size of people of each province in China 1
Table S2. Prevalence of common symptoms and functional limitations after COVID-19 infection assessed by the C19-YRSm, by the most current COVID-19 infection time. 2
Table S3. Prevalence of common symptoms and functional limitations after COVID-19 infection assessed by the C19-YRSm, by COVID-19 infection times. 3
Table S4. Prevalence of common symptoms and functional limitations after COVID-19 infection assessed by the C19-YRSm, by gender. 4
Table S5. Prevalence of common symptoms and functional limitations after COVID-19 infection assessed by the C19-YRSm, by age. 5
Table S6. Prevalence of the other 25 symptoms after COVID-19 infection assessed by the C19-YRSm, by the most recent COVID-19 infection time. 6
Table S7. Prevalence of the other 25 symptoms after COVID-19 infection assessed by times of COVID-19 infection. 7
Table S8. Prevalence of the other 25 symptoms after COVID-19 infection assessed by gender. 8
Table S9. Prevalence of the other 25 symptoms after COVID-19 infection assessed by age. 9
Table S10. The mean severity of 10 common symptoms and 5 functional abilities assessed by the C19-YRSm, scored from 0 to 3, before/after COVID-19 infection. 10
Table S11. The mean severity of 10 common symptoms and 5 functional abilities assessed by the C19-YRSm, scored from 0 to 3, by the most recent COVID-19 infection time. 10
Table S12. The mean severity of 10 common symptoms and 5 functional abilities assessed by the C19-YRSm, scored from 0 to 3, by gender. 11
Table S13. The mean severity of 10 common symptoms and 5 functional abilities assessed by the C19-YRSm, scored from 0 to 3, by times of COVID-19 infection. 11
Table S14. The mean severity of 10 common symptoms and 5 functional abilities assessed by the C19-YRSm, scored from 0 to 3, by age. 12
Table S15. Correlation between scores for symptom severity, functional limitation, other symptoms, and overall health assessed by the C19-YRSm. 13
Table S16. Main effect analysis results of mixed logit model with subgroup analysis by age. 14
Table S17. Main effect analysis results of mixed logit model with subgroup analysis by times of COVID-19 infection. 15
Table S18. Main effect analysis results of mixed logit model with subgroup analysis by the most current infection time. 16
Figure S1. Example of a choice set in the discrete choice experiment. 17
Figure S2. Heat plot of correlation of scores for common symptoms, functional abilities, other symptoms, and overall health measured by the C19-YRSm. Red indicates a positive correlation and blue indicates a negative correlation, with the darker colors indicating a stronger correlation.17

Table S1. Sample proportion and sample size of people of each province in China

Province	Proportion (%)	Number
Beijing	1.55	47
Tianjin	0.98	30
Hebei Province	5.29	159
Shanxi Province	2.48	74
Inner Mongolia Autonomous Region	1.71	51
Liaoning Province	3.02	91
Jilin Province	1.71	51
Heilongjiang Province	2.26	68
Shanghai	1.76	53
Jiangsu Province	6.01	180
Zhejiang Province	4.58	137
Anhui Province	4.33	130
Fujian Province	2.95	88
Jiangxi Province	3.21	96
Shandong Province	7.20	216
Henan Province	7.05	211
Hubei Province	4.10	123
Hunan Province	4.71	141
Guangdong Province	8.94	268
Guangxi Zhuang Autonomous Region	3.56	107
Hainan Province	0.72	21
Chongqing	2.27	68
Sichuan Province	5.94	178
Guizhou Province	2.74	82
Yunnan Province	3.35	100
Xizang Autonomous Region	0.26	8
Shaanxi Province	2.80	84
Gansu Province	1.77	53
Qinghai Province	0.42	13
Ningxia Hui Autonomous Region	0.51	15
Xinjiang Uygur Autonomous Region	1.83	55
Total	100.00	3000

Table S2. Prevalence of common symptoms and functional limitations after COVID-19 infection assessed by the C19-YRSm, by the most current COVID-19 infection time.

C19-YRSm items	Total, n (%)	≤6 months		7-12 months		> 12 months	
		n	Prevalence (%)	n	Prevalence (%)	n	Prevalence (%)
Breathlessness							
At rest	1114 (37.87)	396	40.91	605	34.89	113	47.08
Changing position e.g. from lying to sitting or sitting to lying	1199 (40.75)	399	41.22	671	38.70	129	53.75
On dressing yourself	651 (22.13)	215	22.21	354	20.42	82	34.17
On walking up a flight of stairs	1911 (64.96)	666	68.80	1094	63.09	151	62.92
Cough/throat sensitivity/voice change							
Cough/throat sensitivity	1534 (52.14)	539	55.68	864	49.83	131	54.58
Change of voice	805 (27.36)	277	28.62	447	25.78	81	33.75
Fatigue	2138 (72.67)	731	75.52	1222	70.47	185	77.08
Smell/taste							
Altered smell	915 (31.10)	305	31.51	517	29.82	93	38.75
Altered taste	876 (29.78)	289	29.86	495	28.55	92	38.33
Pain/discomfort							
Chest pain	923 (31.37)	297	30.68	534	30.80	92	38.33
Joint pain	1039 (35.32)	342	35.33	601	34.66	96	40.00
Muscle pain	1140 (38.75)	388	40.08	645	37.20	107	44.58
Headache	1217 (41.37)	421	43.49	685	39.50	111	46.25
Abdominal pain	633 (21.52)	201	20.76	366	21.11	66	27.50
Cognition							
Problems with concentration	1619 (55.03)	560	57.85	916	52.83	143	59.58
Problems with memory	1685 (57.27)	579	59.81	980	56.52	126	52.50
Problems with planning	1209 (41.09)	415	42.87	686	39.56	108	45.00
Palpitations/dizziness							
Palpitations	1353 (45.99)	454	46.90	773	44.58	126	52.50
Dizziness	1548 (52.62)	504	52.07	908	52.36	136	56.67
Post-exertional malaise (worsening of symptoms)	1236 (42.01)	412	42.56	715	41.23	109	45.42
Anxiety/mood	-	670	69.21	1138	65.63	174	72.50
Feeling anxious	1646 (55.95)	546	56.40	958	55.25	142	59.17
Feeling depressed	984 (33.45)	333	34.40	548	31.60	103	42.92
Having unwanted memories of your illness or time in hospital	870 (29.57)	286	29.55	490	28.26	94	39.17
Having unpleasant dreams about your illness or time in hospital	756 (25.70)	250	25.83	424	24.45	82	34.17
Trying to avoid thoughts or feelings about your illness or time in hospital	875 (29.74)	280	28.93	508	29.30	87	36.25
Sleep problems	1852 (62.95)	635	65.60	1053	60.73	164	68.33
Difficulty with communication	825 (28.04)	280	28.93	455	26.24	90	37.50
Difficulties with walking or moving around	573 (19.48)	193	19.94	317	18.28	63	26.25
Difficulties with personal care	284 (9.65)	88	9.09	157	9.05	39	16.25
Difficulty with other activities of daily living	913 (31.03)	298	30.79	521	30.05	94	39.17
Problems with social role	811 (27.57)	254	26.24	472	27.22	85	35.42

Table S3. Prevalence of common symptoms and functional limitations after COVID-19 infection assessed by the C19-YRSm, by COVID-19 infection times.

C19-YRSm items	Total, n (%)	1 time (n=2423)		≥2 times (n=519)	
		n	Prevalence (%)	n	Prevalence (%)
Breathlessness					
At rest	1114 (37.87)	874	36.07	240	46.24
Changing position e.g. from lying to sitting or sitting to lying	1199 (40.75)	996	41.11	203	39.11
On dressing yourself	651 (22.13)	542	22.37	109	21.00
On walking up a flight of stairs	1911 (64.96)	1557	64.26	354	68.21
Cough/throat sensitivity/voice change					
Cough/throat sensitivity	1534 (52.14)	1233	50.89	301	58.00
Change of voice	805 (27.36)	653	26.95	152	29.29
Fatigue	2138 (72.67)	1727	71.28	411	79.19
Smell/taste					
Altered smell	915 (31.10)	749	30.91	166	31.98
Altered taste	876 (29.78)	718	29.63	158	30.44
Pain/discomfort					
Chest pain	923 (31.37)	759	31.32	164	31.60
Joint pain	1039 (35.32)	852	35.16	187	36.03
Muscle pain	1140 (38.75)	924	38.13	216	41.62
Headache	1217 (41.37)	1000	41.27	217	41.81
Abdominal pain	633 (21.52)	526	21.71	107	20.62
Cognition					
Problems with concentration	1619 (55.03)	1321	54.52	298	57.42
Problems with memory	1685 (57.27)	1374	56.71	311	59.92
Problems with planning	1209 (41.09)	988	40.78	221	42.58
Palpitations/dizziness					
Palpitations	1353 (45.99)	1098	45.32	255	49.13
Dizziness	1548 (52.62)	1269	52.37	279	53.76
Post-exertional malaise (worsening of symptoms)	1236 (42.01)	995	41.06	241	46.44
Anxiety/mood					
Feeling anxious	1646 (55.95)	1352	55.80	294	56.65
Feeling depressed	984 (33.45)	799	32.98	185	35.65
Having unwanted memories of your illness or time in hospital	870 (29.57)	717	29.59	153	29.48
Having unpleasant dreams about your illness or time in hospital	756 (25.70)	621	25.63	135	26.01
Trying to avoid thoughts or feelings about your illness or time in hospital	875 (29.74)	721	29.76	154	29.67
Sleep problems	1852 (62.95)	1496	61.74	356	68.59
Difficulty with communication	825 (28.04)	671	27.69	154	29.67
Difficulties with walking or moving around	573 (19.48)	469	19.36	104	20.04
Difficulties with personal care	284 (9.65)	244	10.07	40	7.71
Difficulty with other activities of daily living	913 (31.03)	752	31.04	161	31.02
Problems with social role	811 (27.57)	671	27.69	140	26.97

Table S4. Prevalence of common symptoms and functional limitations after COVID-19 infection assessed by the C19-YRSm, by gender.

C19-YRSm items	Total, n (%)	Male (n=1185)		Female (n=1757)	
		n	Prevalence (%)	n	Prevalence (%)
Breathlessness					
At rest	1114 (37.87)	414	34.94	700	39.84
Changing position e.g. from lying to sitting or sitting to lying	1199 (40.75)	437	36.88	762	43.37
On dressing yourself	651 (22.13)	246	20.76	405	23.05
On walking up a flight of stairs	1911 (64.96)	706	59.58	1205	68.58
Cough/throat sensitivity/voice change					
Cough/throat sensitivity	1534 (52.14)	608	51.31	926	52.70
Change of voice	805 (27.36)	335	28.27	470	26.75
Fatigue	2138 (72.67)	836	70.55	1302	74.10
Smell/taste					
Altered smell	915 (31.10)	386	32.57	529	30.11
Altered taste	876 (29.78)	364	30.72	512	29.14
Pain/discomfort					
Chest pain	923 (31.37)	348	29.37	575	32.73
Joint pain	1039 (35.32)	417	35.19	622	35.40
Muscle pain	1140 (38.75)	450	37.97	690	39.27
Headache	1217 (41.37)	442	37.30	775	44.11
Abdominal pain	633 (21.52)	230	19.41	403	22.94
Cognition					
Problems with concentration	1619 (55.03)	628	53.00	991	56.40
Problems with memory	1685 (57.27)	635	53.59	1050	59.76
Problems with planning	1209 (41.09)	476	40.17	733	41.72
Palpitations/dizziness					
Palpitations	1353 (45.99)	492	41.52	861	49.00
Dizziness	1548 (52.62)	567	47.85	981	55.83
Post-exertional malaise (worsening of symptoms)	1236 (42.01)	454	38.31	782	44.51
Anxiety/mood					
Feeling anxious	1646 (55.95)	629	53.08	1017	57.88
Feeling depressed	984 (33.45)	367	30.97	617	35.12
Having unwanted memories of your illness or time in hospital	870 (29.57)	365	30.80	505	28.74
Having unpleasant dreams about your illness or time in hospital	756 (25.70)	312	26.33	444	25.27
Trying to avoid thoughts or feelings about your illness or time in hospital	875 (29.74)	363	30.63	512	29.14
Sleep problems	1852 (62.95)	718	60.59	1134	64.54
Difficulty with communication	825 (28.04)	333	28.10	492	28.00
Difficulties with walking or moving around	573 (19.48)	227	19.16	346	19.69
Difficulties with personal care	284 (9.65)	131	11.05	153	8.71
Difficulty with other activities of daily living	913 (31.03)	359	30.30	554	31.53
Problems with social role	811 (27.57)	307	25.91	504	28.69

Table S5. Prevalence of common symptoms and functional limitations after COVID-19 infection assessed by the C19-YRSm, by age.

C19-YRSm items	Total, n (%)	<30 (n=1390)		30-34 (n=790)		35-39 (n=421)		≥40 (n=341)	
		n	Prevalence (%)	n	Prevalence (%)	n	Prevalence (%)	n	Prevalence (%)
Breathlessness									
At rest	1114 (37.87)	504	36.26	314	39.75	169	40.14	127	37.24
Changing position e.g. from lying to sitting or sitting to lying	1199 (40.75)	563	40.50	351	44.43	163	38.72	122	35.78
On dressing yourself	651 (22.13)	303	21.80	178	22.53	84	19.95	86	25.22
On walking up a flight of stairs	1911 (64.96)	931	66.98	536	67.85	249	59.14	195	57.18
Cough/throat sensitivity/voice change									
Cough/throat sensitivity	1534 (52.14)	693	49.86	437	55.32	230	54.63	174	51.03
Change of voice	805 (27.36)	352	25.32	230	29.11	116	27.55	107	31.38
Fatigue	2138 (72.67)	1007	72.45	597	75.57	303	71.97	231	67.74
Smell/taste									
Altered smell	915 (31.10)	406	29.21	267	33.80	126	29.93	116	34.02
Altered taste	876 (29.78)	378	27.19	248	31.39	136	32.30	114	33.43
Pain/discomfort									
Chest pain	923 (31.37)	425	30.58	274	34.68	109	25.89	115	33.72
Joint pain	1039 (35.32)	459	33.02	302	38.23	146	34.68	132	38.71
Muscle pain	1140 (38.75)	531	38.20	324	41.01	153	36.34	132	38.71
Headache	1217 (41.37)	597	42.95	344	43.54	155	36.82	121	35.48
Abdominal pain	633 (21.52)	318	22.88	169	21.39	75	17.81	71	20.82
Cognition									
Problems with concentration	1619 (55.03)	814	58.56	444	56.20	201	47.74	160	46.92
Problems with memory	1685 (57.27)	817	58.78	457	57.85	225	53.44	186	54.55
Problems with planning	1209 (41.09)	634	45.61	310	39.24	142	33.73	123	36.07
Palpitations/dizziness									
Palpitations	1353 (45.99)	653	46.98	387	48.99	169	40.14	144	42.23
Dizziness	1548 (52.62)	785	56.47	418	52.91	185	43.94	160	46.92
Post-exertional malaise (worsening of symptoms)	1236 (42.01)	551	39.64	359	45.44	174	41.33	152	44.57
Anxiety/mood									
Feeling anxious	1646 (55.95)	820	58.99	456	57.72	212	50.36	158	46.33
Feeling depressed	984 (33.45)	480	34.53	274	34.68	120	28.50	110	32.26
Having unwanted memories of your illness or time in hospital	870 (29.57)	396	28.49	247	31.27	120	28.50	107	31.38
Having unpleasant dreams about your illness or time in hospital	756 (25.70)	337	24.24	211	26.71	114	27.08	94	27.57
Trying to avoid thoughts or feelings about your illness or time in hospital	875 (29.74)	392	28.20	237	30.00	123	29.22	123	36.07
Sleep problems	1852 (62.95)	891	64.10	521	65.95	254	60.33	186	54.55
Difficulty with communication	825 (28.04)	411	29.57	218	27.59	100	23.75	96	28.15
Difficulties with walking or moving around	573 (19.48)	258	18.56	160	20.25	70	16.63	85	24.93
Difficulties with personal care	284 (9.65)	117	8.42	74	9.37	43	10.21	50	14.66
Difficulty with other activities of daily living	913 (31.03)	428	30.79	260	32.91	113	26.84	112	32.84

Problems with social role	811 (27.57)	393	28.27	230	29.11	86	20.43	102	29.91

Table S6. Prevalence of the other 25 symptoms after COVID-19 infection assessed by the C19-YRSm, by the most recent COVID-19 infection time.

Other symptoms	Total (N=2942)		≤ 6 months (n=968)		7-12 months (n=1734)		> 12 months (n=240)	
	n	Prevalence (%)	n	Prevalence (%)	n	Prevalence (%)	n	Prevalence (%)
Fever	647	21.99	202	20.87	382	22.03	63	26.25
Skin rash/ discolouration of skin	148	5.03	54	5.58	83	4.79	11	4.58
New allergy such as medication, food etc.	99	3.37	35	3.62	53	3.06	11	4.58
Hair loss	601	20.43	203	20.97	346	19.95	52	21.67
Skin sensation (numbness/tingling/itching/nerve pain)	169	5.74	60	6.20	89	5.13	20	8.33
Dry eyes/ redness of eyes	554	18.83	198	20.45	314	18.11	42	17.50
Swelling of feet/ swelling of hands	44	1.50	15	1.55	24	1.38	5	2.08
Easy bruising/ bleeding	56	1.90	18	1.86	28	1.61	10	4.17
Visual changes	159	5.40	47	4.86	100	5.77	12	5.00
Difficulty swallowing solids	218	7.41	62	6.40	139	8.02	17	7.08
Difficulty swallowing liquids	137	4.66	40	4.13	81	4.67	16	6.67
Balance problems or falls	29	0.99	13	1.34	12	0.69	4	1.67
Weakness or movement problems or coordination problems in limbs	385	13.09	111	11.47	241	13.90	33	13.75
Tinnitus	219	7.44	78	8.06	125	7.21	16	6.67
Nausea	244	8.29	87	8.99	134	7.73	23	9.58
Dry mouth/mouth ulcers	240	8.16	82	8.47	140	8.07	18	7.50
Acid Reflux/heartburn	105	3.57	44	4.55	52	3.00	9	3.75
Change in appetite	455	15.47	160	16.53	253	14.59	42	17.50
Unintentional weight loss	125	4.25	54	5.58	51	2.94	20	8.33
Unintentional weight gain	106	3.60	39	4.03	58	3.34	9	3.75
Bladder frequency, urgency or incontinence	71	2.41	29	3.00	36	2.08	6	2.50
Constipation, diarrhoea or bowel incontinence	86	2.92	35	3.62	48	2.77	3	1.25
Change in menstrual cycles or flow	196	6.66	76	7.85	106	6.11	14	5.83
Waking up at night gasping for air (also called sleep apnea)	52	1.77	22	2.27	25	1.44	5	2.08
Thoughts about harming yourself	34	1.16	19	1.96	12	0.69	3	1.25

Table S7. Prevalence of the other 25 symptoms after COVID-19 infection assessed by times of COVID-19 infection.

Other symptoms	Total (N=2942)		1 time (n=2423)		≥2 times (n=519)	
	n	Prevalence (%)	n	Prevalence (%)	n	Prevalence (%)
Fever	647	21.99	546	22.53	101	19.46
Skin rash/ discolouration of skin	148	5.03	117	4.83	31	5.97
New allergy such as medication, food etc.	99	3.37	80	3.30	19	3.66
Hair loss	601	20.43	487	20.10	114	21.97
Skin sensation (numbness/tingling/itching/nerve pain)	169	5.74	135	5.57	34	6.55
Dry eyes/ redness of eyes	554	18.83	439	18.12	115	22.16
Swelling of feet/ swelling of hands	44	1.50	38	1.57	6	1.16
Easy bruising/ bleeding	56	1.90	47	1.94	9	1.73
Visual changes	159	5.40	133	5.49	26	5.01
Difficulty swallowing solids	218	7.41	181	7.47	37	7.13
Difficulty swallowing liquids	137	4.66	113	4.66	24	4.62
Balance problems or falls	29	0.99	23	0.95	6	1.16
Weakness or movement problems or coordination problems in limbs	385	13.09	323	13.33	62	11.95
Tinnitus	219	7.44	177	7.30	42	8.09
Nausea	244	8.29	203	8.38	41	7.90
Dry mouth/mouth ulcers	240	8.16	189	7.80	51	9.83
Acid Reflux/heartburn	105	3.57	81	3.34	24	4.62
Change in appetite	455	15.47	373	15.39	82	15.80
Unintentional weight loss	125	4.25	104	4.29	21	4.05
Unintentional weight gain	106	3.60	81	3.34	25	4.82
Bladder frequency, urgency or incontinence	71	2.41	53	2.19	18	3.47
Constipation, diarrhoea or bowel incontinence	86	2.92	68	2.81	18	3.47
Change in menstrual cycles or flow	196	6.66	155	6.40	41	7.90
Waking up at night gasping for air (also called sleep apnea)	52	1.77	40	1.65	12	2.31
Thoughts about harming yourself	34	1.16	24	0.99	10	1.93

Table S8. Prevalence of the other 25 symptoms after COVID-19 infection assessed by gender.

Other symptoms	Total (N=2942)		Male (n=1185)		Female (n=1757)	
	n	Prevalence (%)	n	Prevalence (%)	n	Prevalence (%)
Fever	647	21.99	286	24.14	361	20.55
Skin rash/ discolouration of skin	148	5.03	59	4.98	89	5.07
New allergy such as medication, food etc.	99	3.37	35	2.95	64	3.64
Hair loss	601	20.43	166	14.01	435	24.76
Skin sensation (numbness/tingling/itching/nerve pain)	169	5.74	66	5.57	103	5.86
Dry eyes/ redness of eyes	554	18.83	196	16.54	358	20.38
Swelling of feet/ swelling of hands	44	1.50	15	1.27	29	1.65
Easy bruising/ bleeding	56	1.90	18	1.52	38	2.16
Visual changes	159	5.40	74	6.24	85	4.84
Difficulty swallowing solids	218	7.41	81	6.84	137	7.80
Difficulty swallowing liquids	137	4.66	52	4.39	85	4.84
Balance problems or falls	29	0.99	7	0.59	22	1.25
Weakness or movement problems or coordination problems in limbs	385	13.09	168	14.18	217	12.35
Tinnitus	219	7.44	91	7.68	128	7.29
Nausea	244	8.29	104	8.78	140	7.97
Dry mouth/mouth ulcers	240	8.16	99	8.35	141	8.03
Acid Reflux/heartburn	105	3.57	50	4.22	55	3.13
Change in appetite	455	15.47	187	15.78	268	15.25
Unintentional weight loss	125	4.25	37	3.12	88	5.01
Unintentional weight gain	106	3.60	41	3.46	65	3.70
Bladder frequency, urgency or incontinence	71	2.41	30	2.53	41	2.33
Constipation, diarrhoea or bowel incontinence	86	2.92	34	2.87	52	2.96
Change in menstrual cycles or flow	196	6.66	-	-	196	11.16
Waking up at night gasping for air (also called sleep apnea)	52	1.77	26	2.19	26	1.48
Thoughts about harming yourself	34	1.16	14	1.18	20	1.14

Table S9. Prevalence of the other 25 symptoms after COVID-19 infection assessed by age.

Other symptoms	Total (N=2942)		<30 (n=1390)		30-34 (n=790)		35-39 (n=421)		≥40 (n=341)	
	n	Prevalence (%)	n	Prevalence (%)	n	Prevalence (%)	n	Prevalence (%)	n	Prevalence (%)
Fever	647	21.99	348	25.04	164	20.76	78	18.53	57	16.72
Skin rash/ discoloration of skin	148	5.03	68	4.89	37	4.68	20	4.75	23	6.74
New allergy such as medication, food etc.	99	3.37	39	2.81	30	3.80	15	3.56	15	4.40
Hair loss	601	20.43	301	21.65	162	20.51	81	19.24	57	16.72
Skin sensation (numbness/tingling/itching/nerve pain)	169	5.74	70	5.04	48	6.08	28	6.65	23	6.74
Dry eyes/ redness of eyes	554	18.83	257	18.49	164	20.76	75	17.81	58	17.01
Swelling of feet/ swelling of hands	44	1.50	12	0.86	14	1.77	10	2.38	8	2.35
Easy bruising/ bleeding	56	1.90	31	2.23	15	1.90	5	1.19	5	1.47
Visual changes	159	5.40	60	4.32	42	5.32	23	5.46	34	9.97
Difficulty swallowing solids	218	7.41	117	8.42	57	7.22	30	7.13	14	4.11
Difficulty swallowing liquids	137	4.66	73	5.25	41	5.19	17	4.04	6	1.76
Balance problems or falls	29	0.99	11	0.79	11	1.39	2	0.48	5	1.47
Weakness or movement problems or coordination problems in limbs	385	13.09	170	12.23	118	14.94	49	11.64	48	14.08
Tinnitus	219	7.44	92	6.62	68	8.61	29	6.89	30	8.80
Nausea	244	8.29	126	9.06	75	9.49	35	8.31	8	2.35
Dry mouth/mouth ulcers	240	8.16	108	7.77	69	8.73	39	9.26	24	7.04
Acid Reflux/heartburn	105	3.57	46	3.31	24	3.04	19	4.51	16	4.69
Change in appetite	455	15.47	235	16.91	123	15.57	62	14.73	35	10.26
Unintentional weight loss	125	4.25	64	4.60	33	4.18	15	3.56	13	3.81
Unintentional weight gain	106	3.60	53	3.81	30	3.80	15	3.56	8	2.35
Bladder frequency, urgency or incontinence	71	2.41	35	2.52	19	2.41	12	2.85	5	1.47
Constipation, diarrhoea or bowel incontinence	86	2.92	36	2.59	25	3.16	19	4.51	6	1.76
Change in menstrual cycles or flow	196	6.66	114	8.20	52	6.58	21	4.99	9	2.64
Waking up at night gasping for air (also called sleep apnea)	52	1.77	22	1.58	13	1.65	9	2.14	8	2.35
Thoughts about harming yourself	34	1.16	18	1.29	9	1.14	3	0.71	4	1.17

Table S10. The mean severity of 10 common symptoms and 5 functional abilities assessed by the C19-YRSm, scored from 0 to 3, before/after COVID-19 infection.

Variables	Total (N=2942)		Now		Pre-COVID	
	Mean	SD	Mean	SD	Mean	SD
Symptom severity						
Breathlessness	1.03	0.78	1.027	0.780	0.629	0.721
Cough/throat sensitivity/voice change	0.77	0.816	0.772	0.816	0.416	0.700
Fatigue	1.01	0.787	1.009	0.787	0.544	0.693
Smell/taste	0.54	0.786	0.539	0.786	0.280	0.632
Pain/discomfort	0.92	0.855	0.918	0.855	0.559	0.780
Cognition	1.05	0.877	1.049	0.877	0.641	0.766
Palpitations/dizziness	0.85	0.836	0.850	0.836	0.497	0.693
Post-exertional malaise	0.57	0.75	0.565	0.750	0.289	0.568
Anxiety/mood	1	0.878	0.997	0.878	0.651	0.766
Sleep	0.9	0.848	0.900	0.848	0.542	0.700
Functional capacity						
Communication	0.36	0.637	0.360	0.637	0.201	0.486
Walking or moving around	0.25	0.546	0.246	0.546	0.154	0.454
Personal care	0.12	0.411	0.124	0.411	0.095	0.366
Other activities of Daily Living	0.38	0.63	0.382	0.630	0.202	0.510
Social role	0.35	0.636	0.355	0.636	0.211	0.517

Table S11. The mean severity of 10 common symptoms and 5 functional abilities assessed by the C19-YRSm, scored from 0 to 3, by the most recent COVID-19 infection time.

Variables	Total (N=2942)		≤6 months (n=968)		7-12 months (n=1734)		>12 months (n=240)	
	Mean	SD	Mean	SD	Mean	SD	Mean	SD
Symptom severity								
Breathlessness	1.03	0.78	1.08	0.767	0.98	0.772	1.19	0.856
Cough/throat sensitivity/voice change	0.77	0.816	0.83	0.83	0.72	0.794	0.88	0.889
Fatigue	1.01	0.787	1.06	0.783	0.97	0.782	1.12	0.817
Smell/taste	0.54	0.786	0.55	0.802	0.52	0.769	0.66	0.833
Pain/discomfort	0.92	0.855	0.94	0.855	0.89	0.841	1.05	0.945
Cognition	1.05	0.877	1.08	0.86	1.02	0.873	1.16	0.959
Palpitations/dizziness	0.85	0.836	0.86	0.841	0.83	0.824	0.98	0.896
Post-exertional malaise	0.57	0.75	0.57	0.749	0.55	0.736	0.67	0.847
Anxiety/mood	1	0.878	1	0.858	0.97	0.874	1.18	0.971
Sleep	0.9	0.848	0.92	0.838	0.86	0.839	1.09	0.917
Functional capacity								
Communication	0.36	0.637	0.37	0.65	0.33	0.615	0.5	0.72
Walking or moving around	0.25	0.546	0.25	0.55	0.23	0.532	0.33	0.618
Personal care	0.12	0.411	0.12	0.402	0.11	0.384	0.23	0.587
Other activities of Daily Living	0.38	0.63	0.39	0.648	0.37	0.615	0.48	0.659
Social role	0.35	0.636	0.33	0.616	0.35	0.63	0.48	0.737

Table S12. The mean severity of 10 common symptoms and 5 functional abilities assessed by the C19-YRSm, scored from 0 to 3, by gender.

Variables	Total (N=2942)		Male (n=1185)		Female (n=1757)	
	Mean	SD	Mean	SD	Mean	SD
Symptom severity						
Breathlessness	1.03	0.78	0.93	0.78	1.09	0.78
Cough/throat sensitivity/voice change	0.77	0.82	0.76	0.83	0.78	0.81
Fatigue	1.01	0.79	0.96	0.78	1.04	0.79
Smell/taste	0.54	0.79	0.56	0.79	0.53	0.78
Pain/discomfort	0.92	0.86	0.87	0.85	0.95	0.86
Cognition	1.05	0.88	1.00	0.87	1.08	0.88
Palpitations/dizziness	0.85	0.84	0.77	0.80	0.91	0.85
Post-exertional malaise	0.57	0.75	0.51	0.73	0.6	0.76
Anxiety/mood	1.00	0.88	0.97	0.88	1.02	0.88
Sleep	0.90	0.85	0.86	0.85	0.92	0.85
Functional capacity						
Communication	0.36	0.64	0.36	0.64	0.36	0.64
Walking or moving around	0.25	0.55	0.24	0.55	0.25	0.55
Personal care	0.12	0.41	0.14	0.41	0.12	0.41
Other activities of Daily Living	0.38	0.63	0.38	0.63	0.39	0.63
Social role	0.35	0.64	0.34	0.63	0.37	0.64

Table S13. The mean severity of 10 common symptoms and 5 functional abilities assessed by the C19-YRSm, scored from 0 to 3, by times of COVID-19 infection.

Variables	Total (N=2942)		1 time (n=2423)		≥2 times (n=519)	
	Mean	SD	Mean	SD	Mean	SD
Symptom severity						
Breathlessness	1.03	0.78	1.02	0.78	1.07	0.79
Cough/throat sensitivity/voice change	0.77	0.82	0.75	0.81	0.86	0.82
Fatigue	1.01	0.79	0.99	0.79	1.09	0.76
Smell/taste	0.54	0.79	0.54	0.78	0.56	0.80
Pain/discomfort	0.92	0.86	0.91	0.86	0.93	0.86
Cognition	1.05	0.88	1.03	0.87	1.12	0.91
Palpitations/dizziness	0.85	0.84	0.84	0.84	0.88	0.83
Post-exertional malaise	0.57	0.75	0.55	0.75	0.62	0.76
Anxiety/mood	1.00	0.88	0.99	0.87	1.04	0.89
Sleep	0.90	0.85	0.88	0.85	0.99	0.85
Functional capacity						
Communication	0.36	0.64	0.36	0.64	0.38	0.65
Walking or moving around	0.25	0.55	0.25	0.55	0.25	0.54
Personal care	0.12	0.41	0.13	0.41	0.11	0.40
Other activities of Daily Living	0.38	0.63	0.38	0.62	0.39	0.65
Social role	0.35	0.64	0.35	0.63	0.36	0.66

Table S14. The mean severity of 10 common symptoms and 5 functional abilities assessed by the C19-YRSm, scored from 0 to 3, by age.

Variables	Total (N=2942)		<30 (n=1390)		30-34 (n=790)		35-39 (n=421)		≥40 (n=341)	
	Mean	SD	Mean	SD	Mean	SD	Mean	SD	Mean	SD
Symptom severity										
Breathlessness	1.03	0.78	1.06	0.79	1.03	0.75	0.99	0.77	0.94	0.83
Cough/throat sensitivity/voice change	0.77	0.82	0.76	0.82	0.81	0.81	0.76	0.79	0.76	0.85
Fatigue	1.01	0.79	1.03	0.81	1.05	0.77	0.96	0.75	0.89	0.76
Smell/taste	0.54	0.79	0.52	0.76	0.55	0.77	0.58	0.85	0.57	0.84
Pain/discomfort	0.92	0.86	0.93	0.86	0.96	0.85	0.85	0.85	0.85	0.86
Cognition	1.05	0.88	1.13	0.90	1.03	0.84	0.9	0.84	0.94	0.89
Palpitations/dizziness	0.85	0.84	0.91	0.86	0.86	0.84	0.72	0.76	0.74	0.80
Post-exertional malaise	0.57	0.75	0.54	0.76	0.59	0.73	0.55	0.74	0.62	0.78
Anxiety/mood	1.00	0.88	1.06	0.90	0.99	0.86	0.9	0.83	0.86	0.88
Sleep	0.90	0.85	0.96	0.90	0.9	0.79	0.81	0.78	0.75	0.82
Functional capacity										
Communication	0.36	0.64	0.39	0.66	0.35	0.62	0.29	0.55	0.37	0.66
Walking or moving around	0.25	0.55	0.24	0.55	0.24	0.52	0.21	0.51	0.33	0.63
Personal care	0.12	0.41	0.11	0.38	0.12	0.40	0.13	0.40	0.20	0.53
Other activities of Daily Living	0.38	0.63	0.38	0.63	0.41	0.64	0.32	0.58	0.40	0.64
Social role	0.35	0.64	0.37	0.66	0.36	0.63	0.27	0.58	0.37	0.62

Table S15. Correlation between scores for symptom severity, functional limitation, other symptoms, and overall health assessed by the C19-YRSm.

	Breathlessness	Cough /throat sensitivity /voice change	Fatigue	Smell /taste	Pain /discomfort	Cognition	Palpitations /dizziness	Post-exertional malaise	Anxiety /mood	Sleep	Communication	Walking or moving around	Personal care	Other activities of daily living	Social role	Symptom severity	Functional capacity	Other symptoms	Overall health
Breathlessness	1.000																		
Cough/throat sensitivity/voice change	0.489	1.000																	
Fatigue	0.572	0.462	1.000																
Smell/taste	0.454	0.487	0.408	1.000															
Pain/discomfort	0.596	0.559	0.565	0.526	1.000														
Cognition	0.578	0.445	0.567	0.436	0.588	1.000													
Palpitations/dizziness	0.589	0.448	0.527	0.432	0.613	0.601	1.000												
Post-exertional malaise	0.491	0.414	0.480	0.416	0.524	0.522	0.549	1.000											
Anxiety/mood	0.540	0.446	0.508	0.428	0.577	0.637	0.576	0.551	1.000										
Sleep	0.468	0.403	0.485	0.357	0.487	0.517	0.505	0.432	0.546	1.000									
Communication	0.390	0.347	0.354	0.385	0.420	0.471	0.411	0.404	0.452	0.374	1.000								
Walking or moving around	0.385	0.343	0.306	0.383	0.433	0.343	0.390	0.399	0.374	0.300	0.366	1.000							
Personal care	0.253	0.294	0.203	0.382	0.348	0.257	0.277	0.282	0.281	0.215	0.373	0.484	1.000						
Other activities of daily living	0.429	0.402	0.388	0.418	0.497	0.442	0.446	0.440	0.475	0.371	0.422	0.455	0.398	1.000					
Social role	0.400	0.375	0.334	0.402	0.456	0.430	0.429	0.407	0.459	0.355	0.455	0.395	0.397	0.475	1.000				
Symptom severity	0.773	0.679	0.745	0.634	0.808	0.796	0.780	0.702	0.781	0.697	0.522	0.467	0.348	0.564	0.532	1.000			
Functional capacity	0.546	0.490	0.472	0.517	0.613	0.583	0.559	0.538	0.598	0.474	0.720	0.633	0.497	0.764	0.735	0.716	1.000		
Other symptoms	0.386	0.345	0.364	0.333	0.423	0.404	0.406	0.366	0.403	0.363	0.304	0.285	0.210	0.344	0.322	0.511	0.428	1.000	
Overall health	-0.302	-0.262	-0.306	-0.232	-0.315	-0.292	-0.272	-0.281	-0.314	-0.272	-0.202	-0.189	-0.132	-0.234	-0.247	-0.389	-0.306	-0.310	1.000

Table S16. Main effect analysis results of mixed logit model with subgroup analysis by age.

Variables	<30 years old				≥ 30 years old			
	Coef.	Std. Err.	z	P-value	Coef.	Std. Err.	z	P-value
Out-of-pocket costs per visit	-0.6100	0.0263	-23.18	<0.001	-0.6432	0.0251	-25.62	<0.001
Hospital level (ref. f primary hospital)								
Tertiary hospital	0.1585	0.0402	3.95	<0.001	0.4130	0.0376	11	<0.001
Medical distance (ref. 20min)								
60min	-1.1769	0.0480	-24.52	<0.001	-1.0924	0.0451	-24.25	<0.001
Therapeutic method (ref. medication-based symptom relief)								
Rehabilitation training	0.2027	0.0552	3.67	<0.001	0.2181	0.0528	4.13	<0.001
Nutrition support	0.2070	0.0606	3.42	0.001	0.3002	0.0557	5.39	<0.001
Medical service type (ref. traditional Chinese medicine)								
Integrated traditional Chinese and Western medicine	0.3174	0.0518	6.12	<0.001	0.1668	0.0497	3.35	0.001
Western medicine	-0.6023	0.0636	-9.47	<0.001	-0.7480	0.0634	-11.8	<0.001
ASC	-0.1183	0.0254	-4.66	<0.001	-0.1052	0.0241	-4.36	<0.001
Number of obs	25020				279936			
Wald $chi^2(7)$	1134.53				1267.61			
Log simulated likelihood	-7037.0110				-7740.5805			
Prob > chi2	0.0000				0.0000			

Note: ASC=Alternative Specific Constant.

Table S17. Main effect analysis results of mixed logit model with subgroup analysis by times of COVID-19 infection.

Variables	1 time				≥2times			
	Coef.	Std. Err.	z	P-value	Coef.	Std. Err.	z	P-value
Out-of-pocket costs per visit	-0.6113	0.0200	-30.53	<0.001	-0.7123	0.0452	-15.75	<0.001
Hospital level (ref. primary hospital)								
Tertiary hospital	0.2610	0.0299	8.74	<0.001	0.4502	0.0693	6.49	<0.001
Medical distance (ref. 20min)								
60min	-1.1479	0.0370	-30.99	<0.001	-1.0871	0.0777	-14.00	<0.001
Therapeutic method (ref. medication-based symptom relief)								
Rehabilitation training	0.2173	0.0419	5.19	<0.001	0.1861	0.0931	2.00	0.046
Nutrition support	0.2548	0.0450	5.66	<0.001	0.2609	0.0999	2.61	0.009
Medical service type (ref. traditional Chinese medicine)								
Integrated traditional Chinese and Western medicine	0.2613	0.0399	6.55	<0.001	0.1320	0.0862	1.53	0.126
Western medicine	-0.6657	0.0492	-13.54	<0.001	-0.7459	0.1107	-6.74	<0.001
ASC	-0.1224	0.0194	-6.32	<0.001	-0.0679	0.0422	-1.61	0.108
Number of obs	43614				9342			
Wald $chi^2(7)$	1856.87				445.03			
Log simulated likelihood	-12216.6570				-2575.6395			
Prob > chi2	0.0000				0.0000			

Note: ASC=Alternative Specific Constant.

Table S18. Main effect analysis results of mixed logit model with subgroup analysis by the most current infection time.

Variables	≤6 months				7-9 months				10-12 months				>12 months			
	Coef.	Std. Err.	z	P-value	Coef.	Std. Err.	z	P-value	Coef.	Std. Err.	z	P-value	Coef.	Std. Err.	z	P-value
Out-of-pocket costs per visit	-0.6714	0.0327	-20.55	<0.001	-0.6176	0.0247	-24.99	<0.001	-0.5849	0.1004	-5.82	<0.001	-0.5510	0.0608	-9.06	<0.001
Hospital level (ref. primary hospital)																
Tertiary hospital	0.3757	0.0496	7.58	<0.001	0.3324	0.0372	8.94	<0.001	-0.0266	0.1370	-0.19	0.846	-0.0998	0.0908	-1.10	0.272
Medical distance (ref. 20min)																
60min	-1.1051	0.0578	-19.12	<0.001	-1.2126	0.0471	-25.76	<0.001	-1.0601	0.1916	-5.53	<0.001	-0.8291	0.1120	-7.40	<0.001
Therapeutic method (ref. medication-based symptom relief)																
Rehabilitation training	0.1579	0.0672	2.35	0.019	0.2365	0.0523	4.52	<0.001	0.5287	0.2121	2.49	0.013	0.1572	0.1186	1.33	0.185
Nutrition support	0.2248	0.0710	3.16	0.002	0.3144	0.0566	5.55	<0.001	0.1923	0.2255	0.85	0.394	0.0714	0.1291	0.55	0.58
Medical service type (ref. traditional Chinese medicine)																
Integrated traditional Chinese and Western medicine	0.1920	0.0624	3.08	0.002	0.3004	0.0503	5.97	<0.001	0.2970	0.2028	1.46	0.143	0.0317	0.1180	0.27	0.788
Western medicine	-0.7202	0.0804	-8.95	<0.001	-0.6296	0.0595	-10.58	<0.001	-0.9573	0.2851	-3.36	0.001	-0.7886	0.1531	-5.15	<0.001
ASC	-0.0641	0.0306	-2.09	0.036	-0.1289	0.0241	-5.34	<0.001	-0.1130	0.0989	-1.14	0.253	-0.1839	0.0582	-3.16	0.002
Number of obs	17424				29448				1764				4320			
Wald $chi^2(7)$	764.67				1168.41				67.36				171.27			
Log simulated likelihood	-4829.9145				-8142.1041				-504.3112				-1280.0272			
Prob > chi2	0.0000				0.0000				0.0000				0.0000			

Note: ASC=Alternative Specific Constant.

If you continue to experience discomfort after COVID-19 infection and seek medical care for long COVID symptoms, which hospital would you choose?

**Attributes**	**Hospital A**	**Hospital B**
Hospital level	Primary hospital	Tertiary hospital
Medical distance	40 min	40 min
Out-of-pocket costs per visit	200 yuan	100 yuan
Therapeutic method	Rehabilitation training	Nutrition support
Medical service type	Western medicine	Integrated traditional Chinese and Western medicine

Figure S1. Example of a choice set in the discrete choice experiment.

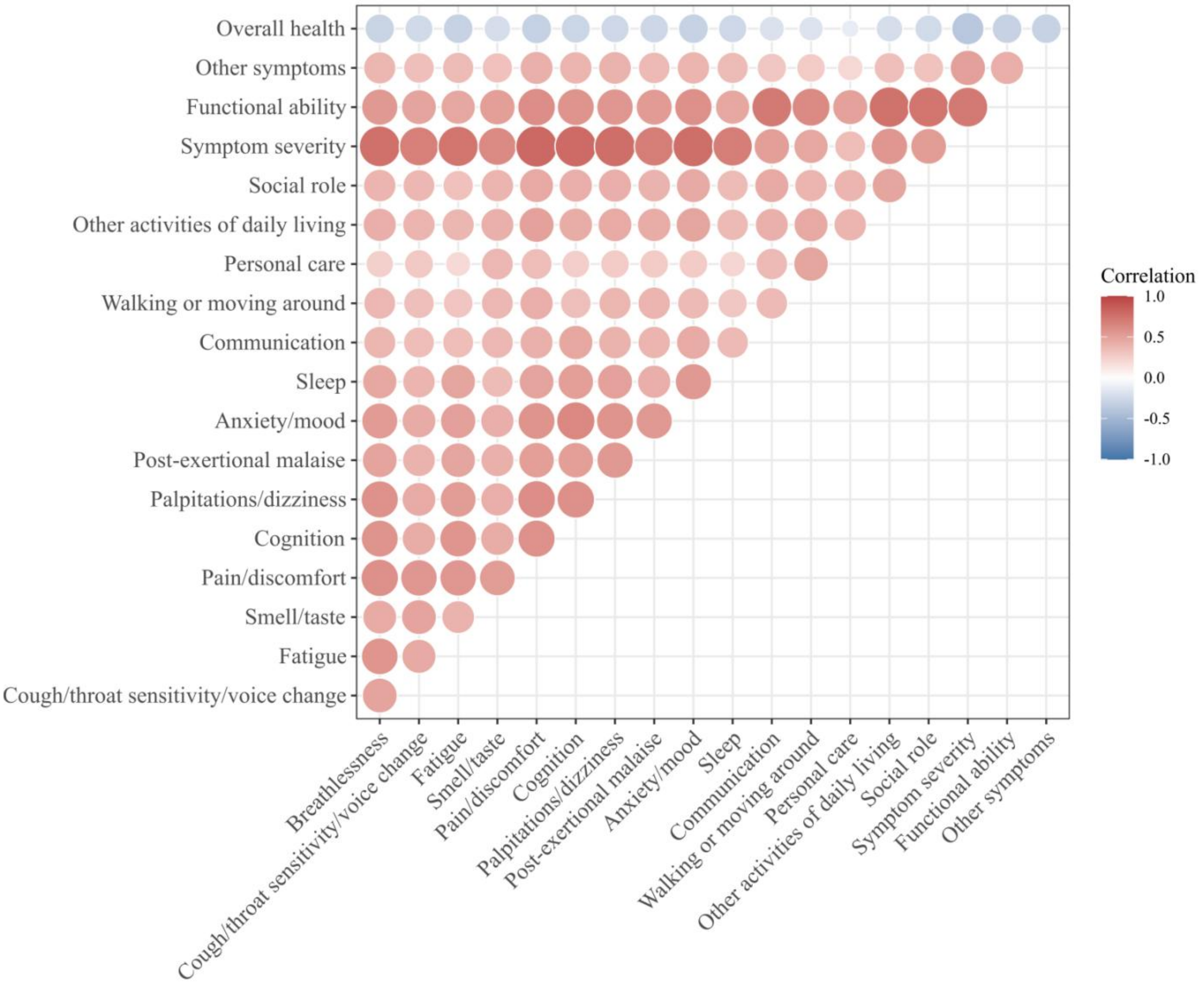

Figure S2. Heat plot of correlation of scores for common symptoms, functional abilities, other symptoms, and overall health measured by the C19-YRSm. Red indicates a positive correlation and blue indicates a negative correlation, with the darker colors indicating a stronger correlation.
